# Employee-Organization Fit and Voluntary Green Behavior: A Cross-Level Model Examining the Role of Perceived Insider Status and Green Organizational Climate

**DOI:** 10.3390/ijerph17072193

**Published:** 2020-03-25

**Authors:** Jincen Xiao, Jih-Yu Mao, Sihao Huang, Tao Qing

**Affiliations:** 1 School of Business Administration, Southwestern University of Finance and Economics, Chengdu 611130, China; xjincen@smail.swufe.edu.cn (J.X.); qingtao@swufe.edu.cn (T.Q.); 2 School of Business, Sichuan University, Chengdu 610065, China; huangsihao@stu.scu.edu.cn

**Keywords:** voluntary green behavior, person-environment fit theory, perceived insider status, green organizational climate, cross-level moderated mediation

## Abstract

Employee green behavior has received considerable attention in recent years because of its contribution to an organization’s environmental performance. However, little is known about how personal and organizational factors can simultaneously affect employee voluntary green behavior. The present study draws on person-environment fit theory to investigate *how* and *when* employee voluntary green behavior can be facilitated by employee-organization fit. Based on a time-lagged survey study of 413 employees from three different manufactures of chemical products, the present study discovers a positive relationship between employee-organization fit and employee voluntary green behavior, and this relationship is mediated by perceived insider status. Moreover, the relationship between perceived insider status and voluntary green behavior is strengthened when employees perceive a green organizational climate. Insights for theory, practice, and future research are also discussed.

## 1. Introduction

One of the most pressing and salient challenges facing organizations today is the urgent need to redress the continuing ecological degradation and strive for a better quality of environment [1,2]. As the notion of “being green makes good business sense” becomes widely accepted, organizational scholars are making more and more endeavors to explore effective ways to promote environmental performance [3,4,5]. However, these studies mostly approached from a firm- or industry-perspective, with little attention paid to intra-organizational processes (e.g., employee behavior) [1,3]. In fact, there is accumulating evidence suggesting that the improvement of an organization’s environmental performance relies a lot on individual employee behaviors [6,7,8,9]. Therefore, in recent years, organizational scholars have shown great interest in investigating employee green behaviors in the workplace [10,11,12].

Employee green behaviors are voluntary or involuntary “scalable actions and behaviors that employees engage in that are linked with and contribute to or detract from environmental sustainability” ([7], p. 87). Since most green behaviors in the workplace are voluntary [12,13] and cryptic in nature [8], voluntary green behavior has received much more scholastic attention than involuntary green behavior, especially pertaining to its antecedents [2,8]. Hitherto, studies in the field of organizational management have mainly adopted three different theoretical perspectives to explore the antecedents of voluntary green behavior. First, based on self-determination theory [14], scholars argued that employees themselves are intrinsically motivated to support environmental sustainability, and they clearly know that they can get rewards and avoid punishment by demonstrating green behavior [15,16]. Second, affective events theory contends that emotional responses bridge work events and behavioral outcomes [17], which suggest that employee voluntary green behavior is to some extent emotionally driven [18]. Third, the exhibition of voluntary green behavior can also be explained by theory of planned behavior [19]. That is to say, voluntary green behavior is not activated by momentary impulses. Rather, it is considered, prepared, and intended [5,20]. Drawing on these theories, empirical studies have mainly identified two categories of antecedents of voluntary green behavior, namely personal factors (e.g., conscientiousness and moral reflectiveness [2]; daily green behavioral attention [5]) and organizational factors (e.g., green human resource management practice [8]; organizational support [21]). However, most these studies focused on either personal factors or organizational factors, while much less is known about their combined effects [22,23].

In order to fill this research gap and expand knowledge of green behavior, this study adopts person-environment fit theory to examine the influences of personal and organizational factors on voluntary green behavior. According to person-environment fit theory, human behaviors are better predicted by the person and the environment together than each of them does separately [24,25]. This theory emphasizes that individuals are more likely to exhibit appropriate behaviors when they are compatible with their environments. Indeed, person-environment fit theory has been used to predict individuals’ positive and discretionary behaviors [26,27]. Employee-organization fit is a type of person-environment fit occurring in an organizational setting. It illustrates the compatibility of individuals and their organizations as well as the alignment of both personal and organizational values [27].

Accordingly, the present study aims to examine *how* and *when* voluntary green behavior can be facilitated by subjective employee-organization fit. Recently, scholars pointed out that employee-organization fit can be interpreted as the psychological state formed by dynamic interactions between employees and organizations [28]. Empirically, employee-organization fit is not only a strong predictor of employee in-role behaviors (e.g., job performance and turnover [29]), but also of extra-role behavior such as organizational citizenship behavior (OCB) [26,27,30]. Since voluntary green behavior is a typical kind of OCB [7,11,13], this study expects that employee-organization fit is a potential antecedent of voluntary green behavior.

Furthermore, the foundation underlying the person-environment fit theory is the psychological mechanism of value congruence [31,32], which fosters employees’ positive states [11,33,34]. Indeed, previous studies have identified psychological states such as psychological ownership [26], satisfaction of needs [35], and organizational commitment [30] as mediators of the relationships between employee-organization fit and work behaviors. Fit with the organization largely induces feelings of connection, which are likely to motivate employees to undertake extra-role actions [36,37]. As such, the present study argues that perceived insider status can play a mediating role in the relationship between employee-organization fit and voluntary green behavior [38]. In addition, person-environment fit theory contends that people have innate needs to fit in their environments, which stimulate appropriate behaviors [33]. Therefore, this study theorizes perceived green organizational climate as a moderating variable, which refers to employees’ shared perceptions of the organization’s pro-environment policies, procedures, and practices [8]. That is, employees who perceive an insider status are likely to be more compatible with their organizations and are more willing to contribute to the organization’s environmental performance by demonstrating green behavior when perceiving a green organizational climate.

**Figure 1:**
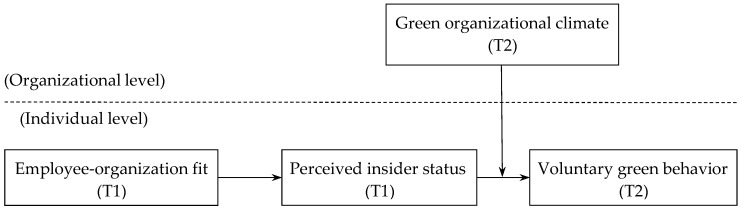
The proposed model of current research. Note: T1 means the data were collected at Time 1, T2 means the data were collected at Time 2.

By testing these hypotheses in a time-lagged study, our investigation of employee-organization fit as an antecedent of employee voluntary green behavior offers several contributions to the literature. First, this study provides novel theoretical insights for relevant environmental research by adopting an employee-centric perspective, especially given that current scholarship is dominated by studies concerning industrial level phenomena [1]. Second, the present study extends knowledge of voluntary green behavior by examining a promising antecedent through the lens of person-environment theory. Specifically, employees are likely to exhibit voluntary green behavior when they fit in with their organizations. This investigation can enrich research on voluntary green behavior because previous studies lack a simultaneous investigation of personal and organizational influences. Third, this study further explores how and when employee-organization fit facilitates voluntary green behavior. That is, employee-organization fit can positively affect perceived insider status, which subsequently leads to voluntary green behavior. Moreover, this study identifies a boundary condition of the relationship between perceived insider status and voluntary green behavior. The present study suggests that the relationship is likely to be more salient when employees perceive a strong green organizational climate. Figure 1 presents the proposed research model.

## 2. Literature Review and Hypothesis Development

### 2.1. Person-Environment Fit Theory and Voluntary Green Behavior

Person-environment fit theory is one of the most compelling frameworks in the field of organizational psychology, and it serves as a critical conceptual foundation for understanding optimal behaviors at work [39,40]. In general, person-environment fit refers to “the degree of match between the values, norms, and other attributes held by both the person and the environment” ([41], p. 172). Person-environment fit theory includes three main principles. First, people are innately motivated to seek out environments that fit themselves or to adjust themselves to fit with the environment. Second, human behaviors are better predicted by the person and the environment together than each of them does separately. Third, optimal outcomes are achieved when personal and environmental attributes are compatible with each other [25]. In addition, employee-organization fit is a type of supplementary fit, which depicts the instance where employees’ attributes are similar to those of organizations [42]. Compared to complementary fit, the occasion in which employees’ attributes are complemented by their organizational environments [24], supplementary fit can better explain inducement of voluntary green behavior because it assumes that humans have a strong, universal, and automatic tendency to seek similarity with others [15,20]. Employee-organization fit is therefore defined as similarities between employees and organizations in their personality, values, or goals [35].

Personal values refer to “abstract beliefs about desirable, trans-situational goals that serve as guiding principles in people’s lives” ([43], p. 111). Value congruence allows employees to achieve fulfillment from their work roles and promote extra-role behaviors [31]. Further, since value is fundamental and relatively enduring, value congruence can be seen as a kind of self-motivation schema that guides employee behaviors [31]. In this regard, it is important to understand employee voluntary green behavior from the perspective of value congruence. Based on supplies-values fit (i.e., a type of person-environment fit), scholars operationalized green human resource management as organizational supplies and regarded individual personality as needs [8]. They found that green human resource management indirectly influenced extra-role green behavior. Although their work did not directly focus on employee-organization fit, they provided preliminary understanding of the relationship between employee-organization fit and voluntary green behavior.

Drawing on person-environment theory, the current study argues that employees who fit in with their organizations are likely to have similar values with their organizations, which in turn, motivate them to exhibit certain behaviors [25]. First, employees who fit in with their organizations usually have positive job attitudes (e.g., organizational commitment) and enable them to hold a strong desire to grow with the organization in the long-run [2,8,31]. Second, employees who perceive higher fit with the organization are more likely to identify with and internalize the organization’s values [31,44,45]. As mentioned before, voluntary green behavior is a kind of pro-environmental behavior that is beneficial for the organization’s environmental performance [5]. Since the society would expect and applaud an organization’s eco-friendly practices, those who have a higher fit with their organizations are more likely to promote, maintain, and defend the organization’s image [36], which drives them to show more voluntary green behavior to sustain such positive image. In other words, employees who fit in with their organizations are more likely to respond to the organization’s eco-friendly practices.

Furthermore, research on OCB also shed light on the relationship between employee-organization fit and voluntary green behavior. Originally, voluntary green behavior was regarded as an environmental OCB that was not explicitly recognized by the formal reward system, despite its contribution to the effectiveness of environmental management [11]. In the field of OCB research, the positive relationship between employee-organization fit and OCB has been well documented [13,19,40], indicating that discretionary work behaviors are most likely to occur when employees perceive higher fit with the organization. In other words, employees who fit in with the organization are more willing to engage in actions on behalf of their organizations [30] and show support for organizational performance [11]. This study thus proposes that employee-organization fit is positively associated with voluntary green behavior, which aligns with the notion of “fitting in and doing good” ([27], p. 353). This leads to the first hypothesis:

**Hypothesis** **1.** *Employee-organization fit is positively related to voluntary green behavior*.

### 2.2. The Mediating Role of Perceived Insider Status

As suggested by person-environment fit theory, the compatibility of individual and organizational values engenders individual psychological reactions which underlie the relationship between employee-organization fit and individual outcome behaviors [25,27]. This notion is supported by two meta-analytic reviews. First, [44] found that employee-organization fit has significant impact on employees’ psychological states such as job satisfaction, organizational commitment, and intent to turnover. [29] extended this research by meta-analytically examining the relationship between employee-organization fit and behavioral outcomes. They argued that employee-organization fit influences individual behavioral outcomes (e.g., job, performance, OCB, turnover) through means of influencing their psychological reactions. Since perceived insider status is an overall evaluation of the relationship between employees and the organization, it is a form of positive psychological state that is likely to be a result of value congruence [36,38]. In other words, employees are likely to thrive and be dedicated to organizations in which their values are also important to the organization [40]. The present study thus argues that perceived insider status plays a mediating role between the relationship of employee-organization fit and voluntary green behavior.

Perceived insider status captures feelings of belongingness in an organization. That is, employees who perceive themselves as insiders are likely to develop personal bond with the organization, which enables them to freely share relevant needs and feelings and generate high level of intimacy [46], regardless of their organizational tenure and positions [47,48]. Existing research has suggested that employees who perceive fit with their organizations often feel belonged and committed [44,45]. Moreover, value congruence depicts a high relationship quality between employees and the organization, which allows employees to see themselves as insiders [41,46]. These inherent commonalities of employee-organization fit and perceived insider status suggest a positive association between them.

Being an insider comes with certain responsibilities [36,37]. Once employees perceive themselves as insiders, they must behave in certain ways to meet these responsibilities [49,50]. These behaviors are more than likely discretionary in nature [39]. For instance, [36] found that when employees perceive themselves as insiders of their organizations, they also accept citizenship responsibilities, which drive them to demonstrate more OCB. Furthermore, according to social exchange theory, when employees build a positive relationship with their organizations, they tend to engage in behaviors that go beyond the formal requirements of their jobs [51]. Hence, this study expects a positive relationship between perceived insider status and voluntary green behavior.

Existing studies argue that employee-organization fit is positively associated with positive psychological states such as liking and emotional enjoyment because employees would be more comfortable to behave in a manner that is consistent with their true selves [52]. In this regard, employee-organization fit motivates employees to establish positive judgements of their relationships with the organization, which facilitate their perceptions of insider status [38,46]. As a result, they usually hold a higher level of commitment to achieving organizational goals and objectives and exhibiting pro-organizational behaviors such as voluntary green behavior [2,8]. In sum, the present study argues that employees who perceive fit with their organizations are likely to perceive an insider status, which in turn, leads to more voluntary green behavior. Therefore, the present study hypothesizes the following:

**Hypothesis** **2.** *Perceived insider status mediates the positive relationship between employee-organization fit and voluntary green behavior*.

### 2.3. The Moderating Role of Green Organizational Climate 

Scholars from environmental and organizational fields agree that organizational factors are important boundary conditions in the process of demonstrating voluntary green behavior [53,54]. Among many organizational factors, organizational climate, in particular, has garnered much attention [5,8]. Organizational climate is typically described as an aggregation of employee perceptions and interpretations of the organization’s policies, procedures, and practices [55]. Indeed, existing literature has well documented the moderating effect of organizational climate on the relationship between employees’ psychological states and their outcome behaviors [56,57]. In particular, green organizational climate refers to employee perceptions and interpretations of the organization’s pro-environmental policies, procedures, and practices [8,10]. Empirically, scholars have found a stronger relationship between green behavioral intentions and green behavior the next day when employees perceive a stronger green organizational climate [5]. Hence, this study theorizes green organizational climate as a moderator in the relationship between perceived insider status and voluntary green behavior.

As suggested by person-environment fit theory, individuals strive to fit in with their environments in order to satisfy need for belongingness, maintain control over their lives, and reduce uncertainty [33]. In other words, green organizational climate acts as an informational cue that green behaviors are expected and valued by the organization, therefore guiding employees to exhibit green behavior. For instance, green human resource management practices can facilitate a green organizational climate [8]. A green organizational climate can more strongly affect the voluntary green behavior of those who see themselves as insiders of the organization. Indeed, person-environment fit theory points out that fit with the environment engenders appropriate behaviors [25]. In addition, employees who perceive an insider status tend to feel a stronger obligation to respond to and comply with the organization’s high expectations for green behaviors. Therefore, when perceiving a green organizational climate, employees who consider themselves as insiders are more inclined to internalize the organization’s green values as part of their self-concepts, thus motivating them to exhibit green behaviors in the workplace.

In contrast, when perceiving a weak green organizational climate, employees are inclined to believe that the organization does not expect many green activities from them. Hence, those who see themselves as insiders of the organization are more likely to comply with the organization’s low expectations for green behaviors. As such, the present study hypothesizes the following:

**Hypothesis** **3.** *Perceived green organizational climate moderates the relationship between perceived insider status and voluntary green behavior, such that the relationship is stronger when perceiving a stronger rather than a weaker green organizational climate*.

In sum, this study argues that employees who fit in with their organizations are likely to engage in voluntary green behavior because they see themselves as insiders of the organization. Those who perceive an insider status are more likely to respond to and comply with the organization’s expectations. Hence, they are more likely to exhibit voluntary green behavior when they perceive a stronger green organizational climate. Accordingly, the present study suggests the following moderated mediation hypothesis:

**Hypothesis** **4.** *Perceived green organizational climate moderates the indirect relationship between employee-organization fit and voluntary green behavior via perceived insider status, such that the indirect relationship is stronger when perceiving a stronger rather than a weaker green organizational climate*.

## 3. Methodology

### 3.1. Samples and Procedures

To test the theoretical model, this study conducted a time-lagged survey study in three large chemical enterprises in China because environmental issues can be a large concern for these manufacturers. The time-lagged survey design was employed to minimize common method variance [58,59]. Specifically, the authors asked the human resource managers to hand out the hard copies of the survey to random employees. Employees were told that their participation is voluntary and that their responses would be kept confidential. Two months later, employees who had participated in the time 1 survey were invited to complete the time 2 survey. This study did not involve any human clinical trials or animal experiments.

All procedures performed in this study involving human participants were in accordance with the ethical standards of the institutional research committee and the 1964 Helsinki declaration. Also, the research purpose and design were reviewed and approved by the three participating organizations prior to the data collection. Informed consent was obtained from all individual participants included in the study.

In the time 1 survey, 600 questionnaires (200 for each firm) were distributed and 521 responses were returned (response rate = 86.83%). The time 1 survey measured employee-organization fit, perceived insider status, and employee demographic information. In the time 2 questionnaire, 437 employees (response rate = 83.88%) filled out the questionnaires, which included items to measure perceptions of a green organizational climate and voluntary green behavior. After matching the surveys with a unique identification code, the final sample consisted of 413 employees (119, 158, and 136 from each firm, respectively). Among them, 61.74% were male; 64.16% had obtained at least a bachelor degree; and 71.91% had an organizational tenure of more than 5 years. Their average age was 35.72 (SD = 9.45) years old.

### 3.2. Measures

The present study adopted the translation and back-translation procedure to generate the Chinese version of measurements [60]. A bilingual research assistant translated the original English items into Mandarin, and then another research assistant translated the items back into English. The research team discussed and settled the discrepancies between the original and back-translated versions of the scales, after which the Mandarin survey was finalized. All items were measured using a five-point Likert scale ranging from 1 = “strongly disagree” to 5 = “strongly agree”. All measures are provided in Appendix A.

#### 3.2.1. Employee-Organization Fit

Employee-organization fit was measured using a 3-item scale in the time 1 questionnaire [61]. This scale has been widely used and demonstrated a high reliability [28,35]. An example item was “My organization’s values and culture provide a good fit with the things that I value in life”. The Cronbach’s α is 0.91.

#### 3.2.2. Perceived Insider Status

Perceived insider status was measured by a 6-item scale in the time 1 questionnaire [38]. A sample item was “I feel very much a part of my work organization”. The Cronbach’s α is 0.76.

#### 3.2.3. Green Organizational Climate

This study measured green organizational climate in the time 2 questionnaire using a 5-item scale [5]. An example item was “My organization is concerned with becoming more environmentally friendly”. The Cronbach’s α is 0.91. This study followed existing literature to aggregate individual responses of each firm to the organizational level to measure each organization’s green organizational climate [2,48,62,63]. As suggested by [64], this study computed *r_wg(j)_* to estimate the interrater reliability. The result indicates substantial agreement among individuals (mean *r_wg(j)_* = 0.81; median = 0.83) [65].

#### 3.2.4. Voluntary Green Behavior

Voluntary green behavior was measured using a 6-item scale in the time 2 questionnaire [2]. Compared with general green behavior scale, this scale specifically measures voluntary green behavior in the workplace, which is more appropriate in the present study. A sample item was “I sort recyclable materials into their appropriate bins when other group members do not recycle them”. The Cronbach’s α is 0.88.

#### 3.2.5. Control Variables

Several control variables were included in the analysis [66]. Based on previous empirical studies on voluntary green behavior, this study included five control variables. First, demographic variables would influence individual green behavior [8,12,67]. Hence, the present study controlled for employee gender (1 = female; 2 = male), age (in years), education (1 = junior college or below; 2 = bachelor; 3 = postgraduate), and organizational tenure (in years). This study also controlled for employees’ environmental attitude as it has been identified to positively predict environmental behavior [5,10,68]. Environmental attitude was measured by an 8-item scale [69]. An example item was “For the benefit of the environment we should be prepared to restrict our momentary style of living”. The Cronbach’s α is 0.86.

### 3.3. Analytical Strategy

Since our data reflected a nested structure, this study conducted multilevel path analysis in Mplus 7.4 using the “Cluster” and “Type = Complex” syntaxes [70]. To test the mediation effect, the Monte Carlo resampling method was adopted to derive coefficient estimates and confidence intervals (CIs) at 95% significance level with 20,000 sample repetitions using an online utility (http://www.quantpsy.org) [71]. The Monte Carlo resampling method has been suggested as a superior method than methods that rely on single sample of data (e.g., Sobel test [72]). All independent variables were grand-mean centered prior to the analysis [73]. To test the moderation effect, this study evaluated the effect of the independent variable on the dependent variable at “high” (one standard deviation above the mean) and “low” (one standard deviation below the mean) values of the moderator.

## 4. Results

### 4.1. Confirmatory Factor Analysis

**Figure 2:**
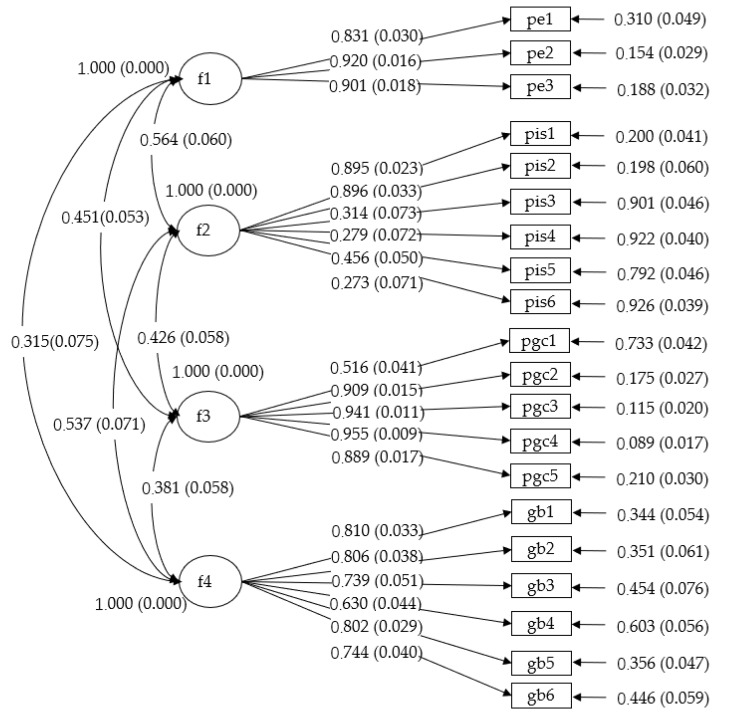
The graphical representation of the measurement model for the hypothesized 4-factor model. *Notes*: f1 = employee-organization fit; f2 = perceived insider status; f3 = green organizational climate; f4 = voluntary green behavior. Values in parentheses are standard error estimates.

This study conducted confirmatory factor analysis to test the measurement model that distinguishes employee-organization fit, perceived insider status, green organizational climate, and voluntary green behavior from one another. As shown in Table 1, the hypothesized 4-factor model fits the data well: *χ^2^* = 328.81, *df* = 164; RMSEA = 0.05; CFI = 0.93; TLI = 0.92; SRMR = 0.01. The hypothesized 4-factor model is superior to any other alternative models. Hence, construct distinctiveness of the main variables is established. In addition, the graphical representation of the measurement model for the hypothesized 4-factor model is shown in Figure 2.

### 4.2. Descriptive Statistics

Means, standard deviations, inter-correlations, and Cronbach’s α are presented in Table 2. The instruments’ construct reliability (CR) and average variance extracted (AVE) are as follows: employee-organization fit (CR = 0.87; AVE = 0.69); perceived insider status (CR = 0.74; AVE = 0.53), green organizational climate (CR = 0.92; AVE = 0.71); and voluntary green behavior (CR = 0.90; AVE = 0.60). These results demonstrate acceptable internal consistencies [74].

### 4.3. Hypothesis Tests

To test the hypotheses, this study simultaneously entered all control variables including employee gender, age, education, organizational tenure, and environmental attitude in the path analysis. The results are presented in Table 3. Hypothesis 1 predicts a positive relationship between employee-organization fit and voluntary green behavior. As shown in Table 3, employee-organization fit is positively related to voluntary green behavior (*B* = 0.20, *SE* = 0.07, *p* < 0.01). Hence, Hypothesis 1 is supported.

Hypothesis 2 argues that perceived insider status mediates the relationship between employee-organization fit and voluntary green behavior. To test the mediation effect, this study first obtained coefficient estimates by regressing perceived insider status on employee-organization fit, and voluntary green behavior on perceived insider status, respectively. As shown in Table 3, employee-organization fit is positively related to perceived insider status (*B* = 0.30, *SE* = 0.03, *p* < 0.001), and perceived insider status is positively related to voluntary green behavior (*B* = 0.33, *SE* = 0.03, *p* < 0.001). These estimates were entered in the Monte Carlo simulation (with 20,000 sample repetitions), and the results indicate that perceived insider status mediates the relationship between employee-organization fit and voluntary green behavior (*indirect effect* = 0.10, *SE* = 0.01, *p* < 0.001, *95% CI* = (0.08, 0.12)). These findings provide support for Hypothesis 2.

**Figure 3:**
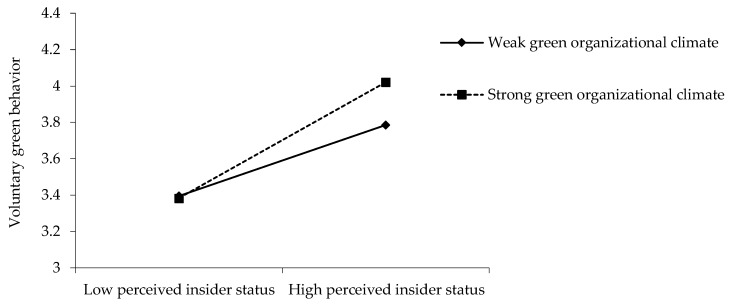
The interactive effect of perceived insider status and green organizational climate on voluntary green behavior.

Hypothesis 3 predicts that green organizational climate moderates the relationship between perceived insider status and voluntary green behavior, such that the relationship is stronger when there exists a stronger rather than a weaker green organizational climate. As shown in Table 3, the results reveal a significant interaction (*B* = 0.17, *SE* = 0.02, *p* < 0.001). Figure 3 illustrates the pattern of this interaction by plotting the simple slopes at “high” (*simple slope* = 0.41, *SE* = 0.03, *p* < 0.001) and “low” (*simple slope* = 0.25, *SE* = 0.03, *p* < 0.001; *difference* = 0.16, *SE* = 0.02, *p* < 0.001) values of green organizational climate. Hence, Hypothesis 3 is supported.

Hypothesis 4 theorizes that green organizational climate moderates the indirect relationship between employee-organization fit and voluntary green behavior via perceived insider status, such that the indirect relationship is stronger when there exists a stronger rather than a weaker green organizational climate. The results as well as the CIs generated from the Monte Carlo simulation (with 20,000 sample repetitions) are presented in Table 4. As indicated, the indirect effect of employee-organization fit on voluntary green behavior via perceived insider status is stronger when green organizational climate is “high” (*indirect effect* = 0.12, *SE* = 0.01, *p* < 0.001, *95% CI* = (0.10, 0.15)), and weaker when it is “low” (*indirect effect* = 0.08, *SE* = 0.01, *p* < 0.001, *95% CI* = (0.05, 0.10); *difference* = 0.05, *SE* = 0.003, *p* < 0.001, *95% CI* = (0.04, 0.06)). Altogether, these results provide support for Hypothesis 4.

## 5. Discussion

To learn more about ways to improve environmental performance, environmental and organizational scholars have increasingly called for nuanced investigation from an employee-centric perspective. This study takes a step forward in this regard. Specifically, this study adopts person-environment fit theory to explore *how* and *when* employee voluntary green behavior can be promoted in the workplace. Using data of 413 employees from three different manufactures of chemical products, this study demonstrates that employee-organization fit has a positive influence on voluntary green behavior, and perceived insider status mediates this relationship. Moreover, green organizational climate moderates the relationship between perceived insider status and voluntary green behavior and the indirect relationship between employee-organization fit and voluntary green behavior via perceived insider status, such that the relationships are strengthened when employees perceive a stronger rather than a weaker green organizational climate. The theoretical and practical implications, limitations, and future research directions are to be discussed.

### 5.1. Theoretical Implications

Our findings extend prior literature in multiple ways. First, this study enriches studies on environmental performance from an employee-centric perspective. Environmental and organizational scholars have raised concerns about the current research which has mostly focused at the industrial level [1,4,7]. For instance, scholars argued that activities that can have an impact on environmental performance are diverse, complex, and therefore difficult to integrate into the formal management systems [75]. Also, these activities rely on employee voluntary contributions, as most employee green behaviors are discretionary and based on good will [6]. As such, organizational scholars have mainly focused on studying employee voluntary green behavior, an extra-role behavior, suggesting that voluntary green behaviors can make significant contributions to an organization’s environmental performance [1]. The present study echoes this view and concentrates on exploring the antecedents of employee voluntary green behavior, which furthers understanding on how intra-organizational influences can facilitate such employee behavior and contribute to environmental performance.

Second, drawing on person-environment fit theory, this study discovers that employees are motivated to exhibit voluntary green behavior when they perceive fit with their organizations. This finding makes significant contribution to the voluntary green behavior literature because existing studies have either investigated its antecedents at the individual or organizational level but have not considered their simultaneous influences. For example, [2] proposed a model of workplace voluntary green behavior in which they only identified personality traits (e.g., conscientiousness and moral reflectiveness) as the antecedents of voluntary green behavior. [8] suggested that green human resource management can cultivate a green organizational climate in organizations, which drives employees to show voluntary green behavior. Although these studies associated voluntary green behavior to a few antecedents, still little is known about the combined influences of personal and organizational factors. Since employee-organization fit involves the interplay of both personal and organizational attributes [28], our study enriches knowledge of the antecedent of voluntary green behavior.

Third, the present study empirically examines how and when employee-organization fit can foster employee voluntary green behavior. Specifically, this study identifies perceived insider status as the linking pin that facilitates this process, which sheds light on how psychological reaction can act as a mediator between employee-organization fit and voluntary green behavior [38,46]. To the best of our knowledge, previous studies have not examined the antecedent role of perceived insider status in predicting voluntary green behavior. In this regard, the present study makes a seminal contribution on how voluntary green behavior can be facilitated. This study also identifies green organizational climate as a boundary condition that affects the extent to which employees who perceive an insider status are likely to engage in voluntary green behavior. The results provide support for the moderation effect, thereby providing novel and interesting insights into how a green climate perception can strengthen or weaken the inducement mechanism of voluntary green behavior. Findings show that when insiders perceive a strong green organizational climate, they are more likely to exhibit voluntary green behavior. In doing so, the present study adopts an interactionist perspective, and further contributes to existing literature by considering how the interplay of individual and organizational determinants can jointly predict individual voluntary behavior [76].

### 5.2. Practical Implications

Since environmental performance has become an important issue for organizational survival [77], employee engagement of voluntary green behavior becomes more and more critical to sustain organizational green practices. Our study provides important implications for managerial practice. First, employee behaviors such as turning off the lights when leaving the office, recycling reusable materials, reducing wastes, and minimizing energy consumptions are considered as voluntary green behaviors in the workplace [5]. Our findings reveal that employee-organization fit has a constructive influence on voluntary green behavior, suggesting that achieving and maintaining employee-organization fit is important for employees to exhibit voluntary green behavior. In this regard, managers should take measures to identify and hire those applicants who fit better with the organization’s values and practices. For example, they can purposefully guide applicants to express their values and personalities during job interviews, based on which they can choose the most suitable applicants for the organization [41].

Second, since findings of this study suggest that perceived insider status plays a mediating role in the relationship between employee-organization fit and voluntary green behavior, managers should pay significant attention to this psychological perception. It is important for managers to be aware of and facilitate subordinate perceptions of an insider status, as it can motivate individuals to engage in citizenship behavior such as green behavior. Also, perceived insider status is a function of perceived positive contributions to the workplace [78]. Managers, acting on the organization’s behalf, can share information and develop personal bond with their employees to foster feelings of an insider status.

Third, prior research has suggested that organizational green policies and practices not only can contribute to environmental performance, but also can enhance organizational competitiveness, increase sales [79], and facilitate brand image and recognition [80]. Therefore, organizations are encouraged to adopt and improve on their green policies and practices to further promote environmental performance. Organizations can instruct and enhance employees’ knowledge, conscience, and awareness of the importance of environmental protection via employee training, organized seminars, and decoration of the physical workplace environment. As evident in our findings, fostering a green organizational climate can be a positive contextual trigger that further stimulates employee voluntary green behavior in the workplace.

### 5.3. Limitations and Future Research Directions

The present study has several limitations that highlight important avenues for future research. First, this study used employee perceptions to measure employee-organization fit. Even if perceived fit is widely used and regarded as the most proximal and strongest predictor of employee behaviors [81,82], other studies have also called for a different measurement of employee-organization fit such as calculated fit. Calculated fit is a more objective measurement of fit, and aims to establish the discrepancy between the employee and organization’s attributes [25]. The lower the discrepancy is, the higher the fit is. Therefore, future studies can adopt this measurement and compare the findings to those of ours in the current study.

Second, scholars have also conceptualized other types of fit in an organizational setting (e.g., employee-job fit, employee-vocational fit, employee-supervisor fit), which have been suggested to influence employees’ psychological experience and behavioral responses [1,2,3]. For example, [3] found that need for competence is satisfied when employees fit in with their jobs, leading to higher job performance. To the full extent of our knowledge, the relationships between these other types of fit and voluntary green behavior have not been well documented. Future research can further explore these relationships and their underlying mechanisms.

Third, our study was conducted in a Chinese context. Hence, cultural influences may have affected our findings. For instance, Chinese employees tend to interpret organizational fit from a relational rather than individualistic perspective [83]. That is, their self-concepts are more likely to be influenced by the larger collective than people in more individualistic societies are [84]. As such, their evaluations of fit may have been affected by the Chinese cultural influence. Moreover, our samples were limited to three manufactures of chemical products, which while well suited for green behavior research, were not inclusive enough. Therefore, the present study encourages future studies to draw samples from different contexts to examine the generalizability of our findings.

In addition, this study urges future scholars to examine other potential mechanisms linking employee-organization fit to voluntary green behavior. For example, existing literature has suggested that higher fit is associated with greater commitment to the organization [24,44]. Employees with high organizational commitment may also voluntarily exhibit green behavior for many reasons such as maintaining the organization’s reputations. This study also encourages future research to investigate other moderating influences. For example, influences from leaders and coworkers are likely to affect the extent to which employees engage in voluntary green behavior. Investigation of their influences can perhaps yield interesting results.

## 6. Conclusions

The present study investigates a multilevel moderated mediation model linking employee-organization fit to voluntary green behavior via perceived insider status. Moreover, the present study highlights the importance of green organizational climate in affecting the relationship between perceived insider status and voluntary green behavior. The multilevel moderated mediation model provides a comprehensive and clear understanding of the antecedents of voluntary green behavior in the workplace. We hope that the theoretical and practical insights gained in this study will advance the understanding of environmental performance from an intra-organizational perspective and inspire researchers to further investigate relevant topics.

## Figures and Tables

**Table 1:** Measurement model fit results.

Models	*χ* *^2^*	*df*	*Δχ^2^*/*df*	RMSEA	CFI	TLI	SRMR
Hypothesized 4-factor model (EOF, PIS, GOC, VGB)	328.81	164	—	0.05	0.93	0.92	0.01
Alternative 3-factor model (EOF + PIS, GOC, VGB)	402.08	167	73.27 *** (3)	0.06	0.90	0.89	0.01
Alternative 3-factor model (EOF, GOC, PIS + VGB)	469.92	167	141.11 *** (3)	0.07	0.88	0.86	0.01
Alternative 3-factor model (EOF, PIS, GOC + VGB)	537.96	167	209.15 *** (3)	0.07	0.85	0.83	0.01
Alternative 2-factor model (EOF + PIS, GOC + VGB)	602.30	169	273.22 *** (5)	0.08	0.82	0.80	0.01
Alternative 2-factor model (EOF + PIS + VGB, GOC)	616.15	169	287.34 *** (5)	0.08	0.81	0.79	0.01
Alternative 1-factor model (EOF + PIS + GOC + VGB)	711.61	170	382.80 *** (6)	0.09	0.78	0.75	0.01

*Notes*: *N* = 413. EOF = employee-organization fit; PIS = perceived insider status; GOC = green organizational climate; VGB = voluntary green behavior; *χ^2^* = chi-square; *df* = degrees of freedom; RMSEA = root mean square error of approximation; CFI = comparative fit index; TLI = Tucker-Lewis index; SRMR = standardized root mean square residual. All models are compared with the hypothesized 4-factor model. *** *p* < 0.001.

**Table 2:** Means, standard deviations, correlations, and reliabilities of studied variables.

Variables	Mean	*SD*	1	2	3	4	5	6	7	8	9
**1. Gender**	1.62	0.49	—								
**2. Age**	35.72	9.45	−0.04	—							
**3. Education**	1.83	0.72	0.05	−0.35 ***	—						
**4. Organizational tenure**	11.22	8.99	−0.02	0.86 ***	−0.36 ***	—					
**5. Environmental attitude**	3.90	0.71	0.01	0.15 **	0.04	0.12 *	(0.86)				
**6. Employee-organization fit**	4.20	0.82	0.04	0.14 **	0.05	0.10 *	0.46 ***	(0.91)			
**7. Perceived insider status**	4.24	0.77	0.12^*^	0.17 ***	0.03	0.21 ***	0.11 *	0.32 ***	(0.76)		
**8. Green organizational climate**	4.17	0.89	0.08	0.10	0.05	0.09	0.22 ***	0.42 ***	0.27 ***	(0.91)	
**9. Voluntary green behavior**	4.38	0.75	0.05	0.02	0.06	0.05	0.24 ***	0.28 ***	0.38 ***	0.28 ***	(0.88)

*Notes*: *N* = 413. SD = standard deviation. Cronbach’s α are presented along the diagonal. Gender: 1 = female; 2 = male. Education: 1 = junior college or below; 2 = bachelor; 3 = postgraduate. * *p* < 0.05, ** *p* < 0.01, *** *p* < 0.001.

**Table 3:** Mplus path analysis results.

Variables	Main Effect	Mediation Effect		Moderation Effect
	Voluntary Green Behavior	Perceived Insider Status	Voluntary Green Behavior	Voluntary Green Behavior
**Intercept**	3.84 *** (0.15)	3.97 *** (0.21)	2.56 *** (0.23)	3.94*** (0.18)
***Control variables***				
**Gender**	0.05 (0.08)	0.17 ** (.06)	-0.01 (0.06)	−0.03 (0.08)
**Age**	−0.01 *** (0.00)	−0.01 (0.01)	-0.01 ** (0.00)	−0.01 ** (0.00)
**Education**	0.05 (0.08)	0.10 * (0.05)	0.01 (0.09)	0.01 (0.10)
**Organizational tenure**	0.01 (0.01)	0.02 *** (0.01)	0.00 (0.00)	0.00 (0.01)
**Environmental attitude**	0.16 * (0.10)	−0.07 *** (0.02)	0.18 * (0.10)	0.18 (0.09)
***Main predictors***				
**Employee-organization fit**	0.20 ** (0.07)	0.30 *** (0.03)	0.10 (0.05)	0.09 (0.06)
**Perceived insider status**			0.33 *** (0.03)	0.33 *** (0.03)
**Green organizational climate**				0.12 * (0.06)
***Interaction***				
**Perceived insider status ×** **Green organizational climate**				0.17 *** (0.02)
**Monte Carlo *95% CI***		(0.08, 0.12)		

*Notes*: *N* = 413. Unstandardized coefficient estimates are reported. Values in parentheses are standard error estimates. Gender: 1 = female; 2 = male. Education: 1 = junior college or below; 2 = bachelor; 3 = postgraduate. * *p* < 0.05, ** *p* < 0.01, *** *p* < 0.001.

**Table 4:** First-stage, second-stage, and conditional indirect effect results.

Outcome	Moderator	Stage		Effect	
	Green Organizational Climate	First (*P*_mx_)	Second (*P*_ym_)	Indirect (*P*_mx_ × *P*_ym_)	95% CI of Indirect Effect
Voluntary green behavior	High (+1 *SD*)	0.30 *** (0.03)	0.41 *** (0.03)	0.12 *** (0.01)	(0.10, 0.15)
	Low (−1 *SD*)	0.30 *** (0.03)	0.25 *** (0.03)	0.08 *** (0.01)	(0.05, 0.10)
	Difference	—	0.16 *** (0.02)	0.05 *** (0.003)	(0.04, 0.06)

*Notes*: *N* = 413. SD = standard deviation. Values in parentheses are standard error estimates. *P*_mx_ = path from employee-organization fit to perceived insider status; *P*_ym_ = path from perceived insider status to voluntary green behavior. 95% confidence intervals (CIs) are derived from the Monte Carlo simulation (with 20,000 sample repetitions) using an online utility [71]. *** *p* < 0.001.

## Appendix A

**Table A1:** Measurement Items.

Variables	Items
Employee-Organization Fit	1. The things that I value in life are very similar to the things that my organization values.
	2. My personal values match my organization’s values and culture.
	3. My organization’s values and culture provide a good fit with the things that I value in life.
Perceived Insider Status	1. I feel very much a part of my work organization.
	2. My work organization makes me believe that I am included in it.
	3. I feel like I am an ‘outsider’ at this organization (R).
	4. I don’t feel included in this organization (R).
	5. I feel I am an ‘insider’ in my work organization.
	6. My work organization makes me frequently feel ‘left-out’ (R).
Green Organizational Climate	The extent to which your company is:
	1. Worried about its environmental impact.
	2. Interested in supporting environmental causes.
	3. Believes it is important to protect the environment.
	4. Concerned with becoming more environmentally friendly.
	5. Would like to be seen as environmentally friendly.
Voluntary Green Behavior	Thinking about your work, to what extent do you…?
	1. Avoid unnecessary printing to save papers.
	2. Use personal cups instead of disposable cups.
	3. Use stairs instead of elevators when going from floor to floor in the building.
	4. Reuse papers to take notes in the office.
	5. Recycle reusable things in the workplace.
	6. Sort recyclable materials into their appropriate bins when other group members do not recycle them.
Environmental Attitude	1. It is still the case that the major part of the population does not act in an environmentally conscious way.
	2. There are limits to economic growth which our industrialized world has crossed or will reach very soon.
	3. Environmental-protection measures should be carried out even if this reduces the number of jobs in the economy.
	4. Thinking about the environmental conditions our children and grandchildren have to live under, worries me.
	5. When I read newspaper articles about environmental problems or view such TV-reports, I am indignant and angry.
	6. If we continue as before, we are approaching an environmental catastrophe.
	7. It is still true that politicians do far too little for environmental protection.
	8. For the benefit of the environment we should be prepared to restrict our momentary style of living

*Notes*: R in the parentheses means reverse code for that item.
